# In Vitro Evaluation of Silica-Coated Bi_2_Se_3_ Nanoplatelets as Photothermal Nanomaterials

**DOI:** 10.1021/acsomega.6c05034

**Published:** 2026-08-05

**Authors:** Blaž Belec, Martina Bergant Marušič, Sebastjan Nemec, Nina Kostevšek, Matjaž Valant

**Affiliations:** † Materials Research Laboratory, 119110 University of Nova Gorica, 5270 Ajdovščina, Slovenia; ‡ Laboratory for Environmental and Life Sciences, University of Nova Gorica, 5000 Nova Gorica, Slovenia; § Department for Nanostructured Materials, “Jožef Stefan” Institute, 1000 Ljubljana, Slovenia; ∥ Jožef Stefan International Postgraduate School, 1000 Ljubljana, Slovenia

## Abstract

Silica-coated Bi_2_Se_3_ nanoplatelets were evaluated in vitro for their photothermal properties and biological response as a more biocompatible alternative to previously reported glycol-coated Bi_2_Se_3_ systems. The assessment was performed using HeLa cells under 808 nm laser irradiation. The silica shell retains the characteristic optical absorption response of Bi_2_Se_3_ and even increases absorption across the UV–vis–NIR spectral range. However, this enhancement is thickness-dependent, as silica layers thicker than ≈50 nm can diminish the optical response of Bi_2_Se_3_. Both thin- and thick-silica-coated nanoplatelets (BiSe@Si-thin and BiSe@Si-thick samples) exhibit pronounced photothermal heating under 808 nm irradiation, reaching 45–52 °C at 0.3 g/L under 1 W/cm^2^ irradiation, corresponding to the subcoagulative hyperthermia regime. This was associated with a significant reduction in cell viability and activation of apoptosis and autophagy pathways. The effect is more pronounced in the BiSe@Si-thin sample, consistent with its higher temperature increase and enhanced cellular association and internalization. No cytotoxicity is observed in the absence of irradiation, confirming that cell death is governed by the photothermal effect.

## Graphical abstract

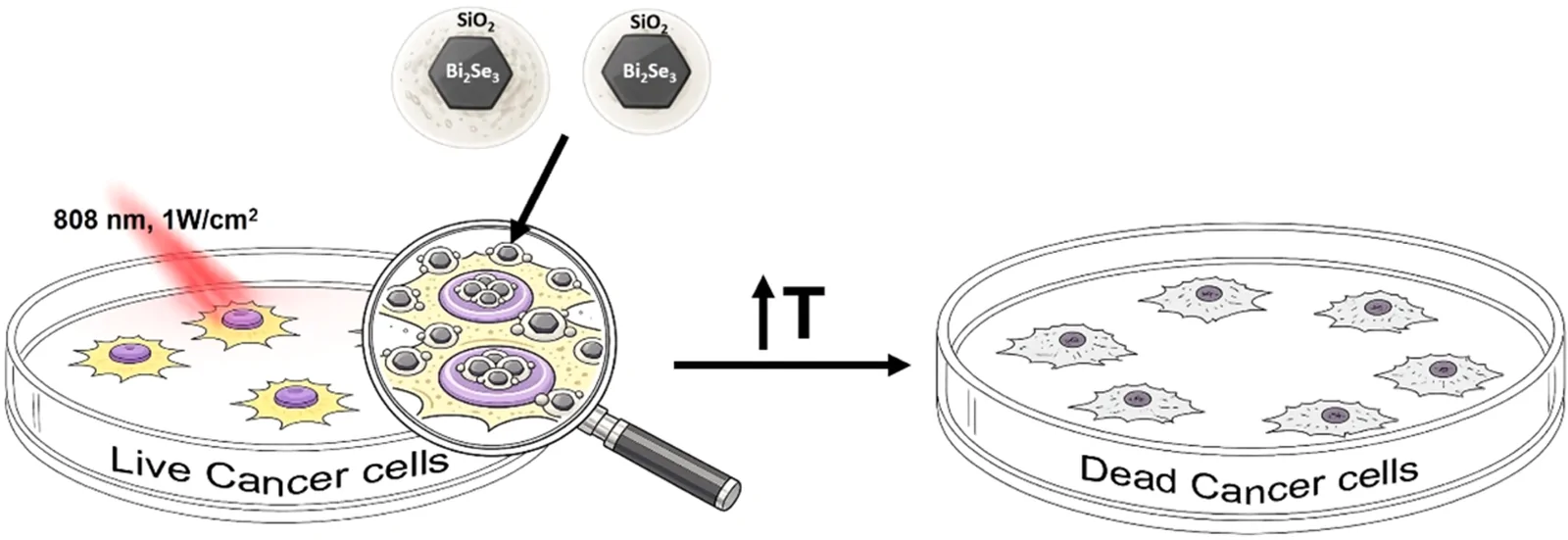

## Introduction

According to the World Health Organization, cancer remains the second leading cause of death worldwide, currently accounting for ≈10 million/year, with incidence projected to reach 12 million deaths annually by 2030.^1^ Despite significant advances in cancer diagnostics and treatment, the increasing mortality rate highlights the urgent need for more effective or entirely new diagnostic and therapeutic strategies. Photothermal therapy (PTT) is a promising approach, offering precise tumor targeting, minimal side effects, and preservation of healthy tissue.^2–5^ The successful clinical application of PTT depends on the choice of material, which ideally must be biocompatible and exhibit strong NIR absorption for efficient heat generation.^6^

Different nanomaterials,^7,8^ including magnetic nanoparticles,^9–18^ carbon nanomaterials,^19,20^ gold-based nanoparticles,^20–25^ and core–shell structured nanoparticles,^7,26–29^ have been explored as possible PT agents. Among emerging material classes, topological insulators (TIs), with bismuth selenide (Bi_2_Se_3_) as a prototypical example, have recently attracted attention for PT-mediated biomedical applications.^6,30–36^ TI are materials that display an interesting electronic structure. They are insulating in the bulk, but host conductive surface states, known as topological surface states (TSS).^37^ Optical excitation induces resonant oscillations of electrons in these states, resulting in localized surface plasmon resonance (LSPR) in the VIS-NIR spectral range and enabling photothermal (PT) effect in TI nanoparticles.^30,38–44^ The suitability of Bi_2_Se_3_ for biomedical applications arises from both its strong PT response and its inherent biocompatibility, linked to the nature of its constituent elements. Bismuth is considered environmentally friendly and, due to its high atomic number, is used as an X-ray contrast agent. In contrast, Se is an essential trace element, with safe daily intake up to 400 μg, known to lower the incidence and fatality of prostate, liver, and lung cancer.^32,36,45^

To date, only a limited number of *in vitro* and *in vivo* studies have explored the use of Bi_2_Se_3_ nanoparticles for PTT, primarily employing ethylene glycol (EG)- or poly­(ethylene glycol) (PEG)-based coatings, with effective concentrations for tumor treatment typically in the range of 0.1–0.3 g/L.^6,30–36^ While these coatings provide sufficient colloidal stability in physiological media, they may introduce certain limitations for biomedical applications. In particular, prolonged exposure may induce immune responses, compromising therapeutic efficacy.^15,25,46–51^ Additionally, glycol coatings reduce light absorption in the VIS and NIR region, directly limiting PT conversion efficiency.^38^

As an alternative, amorphous silica emerged as a highly promising coating due to its biocompatibility, biodegradability, and regulatory approval by both the FDA and EFSA as a nontoxic material, with a safe daily intake of up to 1500 mg/day.^52–54^ Its porous structure and easily modifiable surface further enable versatile functionalization for biomedical applications, such as drug delivery and bioimaging.^52,55–61^ Importantly, silica-coated Bi_2_Se_3_ nanoplatelets exhibit significantly improved biocompatibility^38,56,57,62^ compared to glycol-coated counterparts, while retaining strong VIS-NIR absorption. Moreover, the presence of a silica shell was previously shown to improve PT conversion efficiency under 808 nm irradiation, resulting in up to 2 orders of magnitude higher performance than glycol-coated counterparts and enabling lower therapeutic doses.^38^ While silica-coated nanoparticles, such as FDA-approved iron oxide nanoparticles,^16,63,64^ or the golden standard of PT nanoparticles in biomedicine, Au,^65–67^ have been extensively investigated for PTT in both in vitro and in vivo models, silica-coated Bi_2_Se_3_ nanoparticles have not been explored in this context, despite their demonstrated biocompatibility and excellent PT performance.

In this study, we address this gap by investigating silica-coated Bi_2_Se_3_ nanoplatelets as a potential alternative to previously reported glycol-coated counterparts. Their photothermal performance and in vitro anticancer activity were evaluated in HeLa cells under 808 nm laser irradiation. We investigated cell viability, apoptosis, autophagy, and nanoparticle internalization. Our results show that PT- induced reduction in cell viability was observed at a concentration of 0.3 g/L, reaching temperatures suitable for controlled cell death under the given irradiation conditions. The qualitative internalization investigation results indicate that particle size plays a more important role than surface charge in cellular interaction, with thinner silica-coated nanoplatelets (2–5 nm) showing higher cellular internalization and membrane accumulation than thicker silica-coated nanoplatelets (50–80 nm thick silica layer).

## Methods

### All the Chemicals were Used without Further Purification

#### Bi_2_Se_3_ Nanoparticles Synthesis

Bismuth­(III) nitrate pentahydrate (Bi­(NO_3_)_3_·5H_2_O), bismuth­(III) oxide (Bi_2_O_3_, 99.98%), selenium powder (Se, ≥99.5%), hydrochloric acid (HCl), hydrazine hydrate (N_2_H_4_·H_2_O, 35%), sodium hydroxide (NaOH), ethylene glycol (C_2_H_6_O_2_, 99%), poly­(vinylpyrrolidone) (PVP, MW = 8000).

### All Chemicals were Purchased from Alfa Aesar

#### Amorphous Silica Coating and Labeling

Hexadecyltrimethylammonium bromide (CTAB) was obtained from Alfa Aesar (Kandel, Germany), absolute ethanol and aqueous ammonia (∼25%) from Merck KGaA (Darmstadt, Germany), and cyclohexane from VWR Int. GmbH (Vienna, Austria), tetraethoxysilane (TEOS), (3-aminopropyl)­triethoxysilane, dichloromethane, and concentrated hydrobromic acid (∼48%; HBr) from Sigma-Aldrich (St. Louis, MO, USA). 0.1 M HBr was prepared by diluting concentrated HBr (∼48%) with deionized water. Fluorescein isothiocyanate was obtained from Thermo Fisher Scientific (Waltham, MA, USA).

##### Synthesis of Bi_2_Se_3_ Nanoplatelets

The Bi_2_Se_3_ nanoplatelets were prepared following our previously reported hydrothermal procedure described in ref 68. In short: The stoichiometric amounts of bismuth nitrate and selenium in the molar ratio of 2:3 was dissolved in of deionized water under vigorous stirring, followed by the addition of 11 M HCl and hydrazine. The obtained gray slurry was sealed in a Teflon-lined autoclave and heated at 240 °C for 48 h.^68^ The product was then washed several times with deionized water. After purification, such adsorbent-free nanoplatelets were coated with amorphous SiO_2_ (silica) of different thicknesses. The sample is denoted as “**BiSe**”.

##### Coating of Bi_2_Se_3_ Nanoparticles with Amorphous Silica

The silica-coated nanoplatelets and concentrations employed in this study were selected based on previous biocompatibility assessments, ensuring minimal intrinsic cytotoxicity while preserving the optical response of Bi_2_Se_3_.^38^

#### Thin Silica Coating

A thin (2–5 nm) SiO_2_ coating was deposited onto the BiSe nanoplatelets according to the procedure described in ref 38. Briefly, 320 mg of CTAB was dissolved in 40 mL of deionized H_2_O, with pH adjusted to 3.30 using 0.1 M HBr. Parallelly, 200 mg of dried BiSe sample was dispersed in 18 mL of deionized H_2_O. The formed BiSe suspension was added to the acidic CTAB solution, and the pH of the formed mixture was adjusted to pH 2.85 with 0.1 M HBr. A solution of 2.8 mL of TEOS in 5.2 mL of absolute ethanol was added and whole reaction mixture was stirred for additional 90 at room temperature. After this, the pH was adjusted to 5.0 with aqueous ammonia, and the reaction was left stirred at room temperature overnight. The silica-coated nanoplatelets were washed several times with ethanol and water, and dispersed in water. The sample is denoted as “**BiSe@S-thin**”.

#### Thick Silica Coating

A thick (50–80 nm) SiO_2_ coating was deposited onto the BiSe nanoplatelets according to the procedure described in ref 38. Here BiSe@S-thin nanoplatelets were used as a starting material. Ten mL of the BiSe@S-thin aqueous suspension was added and diluted with 40 mL of deionized water. Then, 12 mL of aqueous ammonia (∼25%) was added. After mixing and sonification, 0.7 mL of TEOS dissolved in 45 mL of absolute ethanol was added to initiate the formation of the silica shell. The reaction mixture was further sonicated for 5 min in an ultrasound bath and stirred overnight. The silica-coated BiSe nanoplatelets with 50–80 nm thick SiO_2_ layer were collected using centrifugation, washed several times with ethanol and water, and dispersed H_2_O. The sample is denoted as “**BiSe@S-thick**”.

##### Preparation of Fluorescent Silica-Coated Bi_2_Se_3_ Nanoparticles

The fluorescent Bi_2_Se_3_ nanoparticles were prepared by coating the core Bi_2_Se_3_ with a layer of silica modified with silane-functionalized fluorescein molecules.

#### Silane-Functionalized Fluorescein

0.5 mg of fluorescein isothiocyanate, 200 μL of dichloromethane, and 5 μL of (3-aminopropyl)­triethoxysilane were mixed in a glass vial. The vial was covered with aluminum foil and left for 1 h at room temperature with occasional hand stirring. Next, 1 mL of absolute ethanol was added to the vial, stirred by hand, and the resulting reaction mixture was used immediately to prepare thin fluorescent silica-coated Bi_2_Se_3_ nanoparticles.

#### Fluorescent Silica Coating

BiSe nanoplatelets coated with thin and thick fluorescent SiO_2_ layer were prepared by the same coating procedure, to obtain thin and thick SiO_2_ layer, as described above and in ref 38, with difference, that in the step of adding TEOS and ethanol, additionally the silane-modified fluorescein was added. The samples were denoted as “**BiSe@SF-thin**” and “**BiSe@SF-thick**”.

### Material Characterization

The synthesized product was characterized using a Rigaku MiniFlex X-ray powder diffractometer with Cu Kα radiation (λ = 1541 Å, 30 kV, 10 mA). For TEM analysis, the platelets were suspended in ethanol and deposited onto a copper grid supported by a lacy carbon film. The TEM analysis was performed using a field-emission electron microscope (JEOL JEM-2100UHR) operating at 200 kV and equipped with an Oxford X-Max80T energy-dispersive X-ray spectroscopy (EDX) detector. The width of the platelets, expressed as an equivalent diameter, was determined from TEM images, on which 200–300 platelets per sample were counted for statistical analysis using Gatan Digital Micrograph Software. The obtained data, representing the frequency count of the size distribution, were fitted using the single- or multiple-peak Gaussian fit mode. The colloidal stability of the platelets in water and biological media (DMEM and DMEM supplemented with 10% FBS) was assessed with electrokinetic (ζ) potential determination using electrophoretic mobility and hydrodynamic particle size with polydispersity index using dynamic light scattering (DLS) on a Litesizer 500 particle analyzer (Anton Paar, Graz, Austria) in a proprietary polycarbonate Omega cuvette (Anton Paar, Graz, Austria). For the samples in water, the nanoplatelets were dispersed in deionized water to a concentration of 0.3 mg/mL and the ζ-potential of the platelets in the aqueous solution was measured as a function of the suspension pH. The pH of the aqueous suspension was adjusted with 0.1 or 0.01 M aqueous NaOH or HCl solution. The pH was measured with a Metrohm 913 pH meter (Metrohm, Herisau, Switzerland) equipped with an NTC temperature-controlled Solitrode pH electrode (Metrohm, Herisau, Switzerland). The hydrodynamic size of the platelets dispersed in water was determined at 5 min, 3 h, and 24 h after dispersion. For determining the ζ-potential and hydrodynamic size of the platelets in biologic media, first the nanoplatelets were dispersed in deionized water to a concentration of 6 mg/mL and further diluted to 0.3 mg/mL in either biological media DMEM or DMEM supplemented with 10% FBS. The ζ-potential and hydrodynamic size of the platelets in biological media were measured at 5 min, 3 h, and 24 h after mixing with the biological media.

The light absorption properties were analyzed with classical UV–vis spectroscopy. The spectroscopy was performed on a PerkinElmer Lambda 950 spectrometer using a quartz cuvette measuring 1 × 1 × 3 cm^3^. A measurement range of λ = 200–850 nm was used with a scanning rate of 1 nm/s. Before the measurements, the platelet suspension was stabilized to the desired ζ-potential and sonicated to break any possible agglomerates.

PT experiments were performed using an FC-808 Fiber Couple Laser System (CNI Optoelectronics Tech, Changchun, China) configured for continuous-wave operation at 808 nm. PT experiments were performed by focusing a continuous-wave 808 nm laser onto the sample using an optical lens positioned at a fixed distance of 5 cm. The samples consisted of water and nanoplatelet suspensions (Bi_2_Se_3_ + silica coating) at concentrations of 0.1 and 0.3 mg/mL, prepared in growth medium. These concentrations were selected based on prior findings indicating negligible effects on cell viability in the absence of irradiation and control (growth medium).^38^ Each well was exposed to laser light at a power density of 1 W/cm^2^ for 5 min. All *in vitro* PT therapy experiments were conducted at this power level, which is commonly used and considered moderate in preclinical PTT studies.^69–73^ The temperature change was tracked with a thermal camera (FLIR E8). Every minute, the temperature was noted. Each experiment was repeated three times.

### In Vitro Evaluation

#### Cell Line

The HeLa (CCL-2) cell line was used for all *in vitro* experiments. The cells were grown at 37 °C in a humidified atmosphere and 5% CO_2_. The growth medium was Dulbecco’s modified Eagle’s medium (DMEM) (Gibco, UK), supplemented with 10% fetal bovine serum (FBS) (Gibco, Germany), 100 μg/mL penicillin, and 100 μg/mL streptomycin (Sigma-Aldrich, Israel). The medium was changed every 2–3 days. When the cells reached 70–90% confluence, they were detached with 0.25% trypsin/EDTA solution (Gibco, UK) and subcultured at a ratio of 1:5.

#### PrestoBlue Viability Assay

HeLa cells were seeded at 2 × 10^4^ cells per well in a 96-well microtiter plate and grown overnight. The cells were then exposed to nanoparticle suspensions of 0.1 and 0.3 mg/mL and incubated for another 24 h. Then they were irradiated with laser light at a power density of 1 W/cm^2^ for 5 min. As negative controls, untreated cells (Untreated control), as well as cells exposed to laser irradiation (Laser) in growth medium without nanoparticles, were used. After irradiation, the cells were transferred to a cell incubator for 4 or 24 h. The cells were then washed with phosphate-buffered saline (PBS) and incubated for 1 h with 10% PrestoBlue Viability Reagent (Invitrogen, OR, USA) in growth medium. The viability of the HeLa cells was determined by measuring the metabolic conversion of the resazurin-based PrestoBlue dye to highly fluorescent resorufin using the Infinite F200 microtiter plate reader (Tecan, Austria) with excitation/emission wavelengths of 560/595 nm. The fluorescence intensities obtained were corrected for background fluorescence and normalized to the laser-irradiated control cells.

#### Apoptosis Assay

HeLa cells were exposed to a nanoparticle suspension exposed to a laser as described above. Apoptosis was analyzed by measuring the enzymatic activity of caspase-3 and caspase-7 24 h after exposure using the Apo-ONE Homogeneous Caspase-3/7 Assay (Promega, USA) according to the manufacturer’s instructions. In brief, the Apo-ONE Caspase-3/7 reagent, containing a fluorescent substrate and cell lysis buffer, was added to the samples in a 1:1 volume ratio. After 2 h of incubation, the amount of fluorescent product was analyzed using an Infinite F200 microplate reader with excitation/emission wavelengths of 485/535 nm. The values obtained were corrected for background fluorescence and normalized to the laser-irradiated control cells.

#### Western Blotting

Cells were exposed as described above and harvested 20 h later to determine the induction of autophagy. Whole cell extracts in RIPA buffer were incubated at 4 °C for 20 min, centrifuged at 10,000*g* for 10 min at 4 °C, and supernatants were recovered. Equal amounts of protein were separated on 12% SDS-polyacrylamide gels and transferred to Hybond ECL nitrocellulose membrane (Amersham, Biosciences, USA). The membranes were then blocked with 1× PBS/0.1% Tween (PBS/T) containing 5% nonfat milk and probed overnight at 4 °C with primary antibodies against LC3 (L8918; Sigma, USA) and α-actinin (H-300, Santa Cruz Biotechnology, USA). They were then washed and incubated for 1 h at room temperature with horseradish peroxidase (HRP)—conjugated secondary antibodies (1:3000; Sigma, USA). The HRP-bound proteins were detected using SuperSignal West Pico PLUS chemiluminescent substrate (Thermo Scientific, USA), and visualization was performed using the Alliance Mini 4W chemiluminiscence imager (UVITEC, UK).

#### Immunofluorescence

HeLa cells were seeded on 9 mm sterile coverslips at a density of 0.5 × 10^5^ cells per well in 12-well plates and grown overnight at 37 °C. BiSe@SF-thin and BiSe@SF-thick nanoparticles at a concentration of 0.2 mg/mL were added to the growth medium, and cells were incubated for another 24 h in the cell incubator. Cells were then rinsed with PBS and immediately fixed with 4% (v/v) paraformaldehyde in PBS for 15 min at room temperature, followed by counterstaining with 4,6-diamidino-2-phenylindole (DAPI) (Sigma). Cell samples were then mounted on glass slides and acquired using an Olympus IX81 microscope. A minimum of 150 cell were analyzed for each experimental condition.

### Statistical Analyses

At least three independent experiments were performed for the assessment of viability and apoptosis, with 2–3 technical replicates in each experiment. Cells were analyzed by one-way ANOVA with Tukey’s multiple comparisons test, comparing treated and control cells. Multiplicity-adjusted P values were calculated for each comparison, where significance was accepted at and shown as *p < 0.05, **p < 0.01, ***p < 0.001, and ****p < 0.0001. Statistical analyses were performed using GraphPad Prism 10 software (GraphPad Software, CA, USA). GraphPad Prism 10 was also used for data calculation, tabulation, and graphical representations.

## Results and Discussion

Figure 1a shows the XRD pattern of hydrothermally synthesized Bi_2_Se_3_ nanoparticles (sample BiSe) nanoplatelets, which were subsequently coated with an amorphous silica layer, compared with the reference Bi_2_Se_3_ card. (JCPDS 33–0215). The BiSe pattern displays peaks that can be indexed according to the Bi_2_Se_3_ rhombohedral crystal structure with space group *R*3*m*. The sharpness of the peaks indicates good crystallinity, while minor broadening suggests nanoscale particle dimensions. As presented in previous articles, hydrothermally synthesized Bi_2_Se_3_ nanoparticles display hexagonal, plate-like morphology (Figure 1b) with a broad size distribution, with a platelet mean diameter of 56 ± 40 nm and a small fraction of bigger particles in the range of 200–300 nm, which resulted from Ostwald ripening.^38,68^ Silica coating of those nanoparticles resulted in a thin ≈2–5 nm (sample BiSe@S-thin) and thick ≈50–80 nm (sample BiSe@S-thick) silica layer (Figure 1d,1e, respectively). In both cases, silica forms a continuous, uniform layer over the BiSe nanoplatelets.

**Figure 1:**
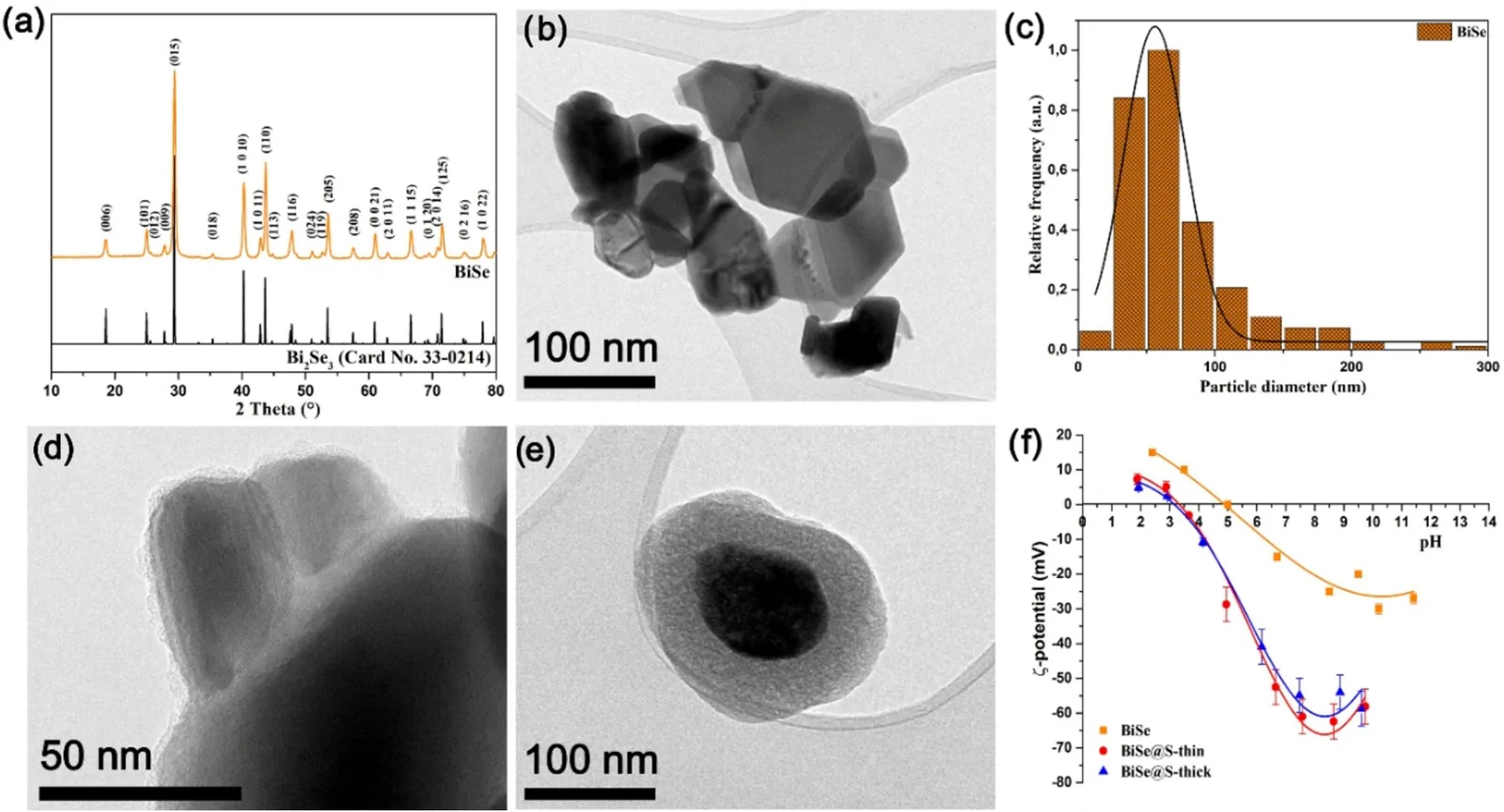
XRD patterns of Bi_2_Se_3_ nanoplatelets used for silica coating, compared with the reference pattern (JCPDS 33–0214) (a). Representative TEM image of the nanoplatelets (b) and the corresponding size distribution (c). Representative TEM images of silica-coated nanoplatelets with a thin (2–5 nm; BiSe@S-thin) (d) and thick (50–80 nm; BiSe@S-thick) silica shell (e). ζ-potential behavior of silica-coated nanoplatelets compared to pristine Bi_2_Se_3_ nanoplatelets (f).

Successful coating of BiSe nanoplatelets with silica is further confirmed by the ζ-potential measurements as a function of pH (Figure 1f). Consistent with previous work,^38^ a clear shift in the point of zero charge (PZC) is observed, decreasing from about pH ≈ 5 for pristine BiSe to ≈3.3 for BiSe@S-thin and ≈3.1 for BiSe@S-thick, which confirms successful silica coating of the BiSe nanoplatelets. The PZC in the range of 2.5–3.5 is characteristic of unmodified silica,^18,46^ which further supports the TEM observations and unequivocally confirms the successful silica coating of the BiSe nanoplatelets. Below the PZC, the ζ-potential shifts to positive due to the formation of positively charged Si–OH_2_^+^ functional groups on the silica surface. Conversely, at pH values greater than the PZC, the ζ values shift to negative, due to the formation of negatively charged Si–O^–^ functional groups on the silica surface. Similarly, in the case of surface-clean Bi_2_Se_3_ nanoplatelets, which have a layered structure terminating in Se^2–^ ions,^47^ the positive ζ-potential under acidic conditions can be attributed to the adsorption of positively charged hydronium ions (H_3_O^+^). At neutral and alkaline pH, the ζ-potential becomes negative due to the adsorption of negatively charged hydroxyl ions (OH^–^).

Additionally, time-dependent colloidal stability of the silica coated BiSe platelets was investigated using DLS and ζ-potential in pure water and biological media. DLS hydrodynamic size measurements show (Figure S1), that both the BiSe@S-thin and BiSe@S-thick silica coated platelets have a wide particle size in pure water ranging from submicron to several μm, indicating a certain degree of agglomeration. However, when determining the hydrodynamic size of the silica-coated BiSe in biological media, their hydrodynamic particle size distribution remains similar to water, which indicates that the colloidal stability of the platelets is retained to an equal degree as in pure water. The detailed measurement data are presented in Supporting Info S1, Figure S1.

The retained colloidal stability of the silica-coated BiSe platelets is further confirmed with ζ-potential measurements (Supporting Info Part S2, Figure S2). Additional ζ-potential measurements were done to further investigate the time-dependent colloidal stability of the silica-coated BiSe platelets in biological media DMEM and DMEM supplemented with 10% FBS. In pure water, the silica-coated BiSe had a mean ζ-potential in the range of negative ∼25 mV (Figure 1f), which indicated good colloidal stability. In biological media, both the thin and thick silica-coated BiSe platelets retained the negative ζ-potential, in the range of −10 to −20 mV in the time frame of up to 24 h (Figure S2). The measurements therefore confirm the colloidal stability is retained in the biological media as well.

An effective PT agent for biomedical applications should exhibit strong optical absorption in the near-infrared (NIR) region. Consistent with previous reports, silica-coated Bi_2_Se_3_ nanoplatelets (BiSe@S-thin and BiSe@S-thick) retain the characteristic optical response of the pristine nanoplatelets, with the addition of a broad absorption band in the ≈200–230 nm spectral region attributed to amorphous silica.^38^ Importantly, the spectral features are preserved in the region >400 nm, which is attributed to the LSPR, responsible for the PT effect.^30^

While the overall spectral profile remains unchanged, the silica-coated samples exhibit a noticeably higher absolute absorption intensity across the entire spectral range compared to the pristine nanoplatelets, indicating enhanced light-harvesting capability. Among the silica-coated samples, BiSe@S-thin exhibits the highest absorption throughout the measured region, whereas BiSe@S-thick shows slightly lower absorption but still exceeds that of the uncoated BiSe. Notably, the UV–vis measurements were performed at equal sample concentrations. Under these conditions, a lower absorption would normally be expected for the silica-coated samples, as the overall Bi_2_Se_3_ nanoplatelet content is reduced by the additional silica mass. Specifically, the Bi_2_Se_3_ fraction is approximately 3.6% and 26.6% lower for BiSe@S-thin and BiSe@S-thick, respectively.^38^ Despite this, the coated samples exhibit increased optical absorption, which can be attributed to improved dispersion stability and reduced light scattering in suspension provided by the silica shell.^74–76^ As shown in Figure 2b, this enhancement is observed only up to a certain silica shell thickness. Beyond this threshold, the optical contribution of silica becomes dominant, leading to decreased absorption across the measured spectral range, as observed for BiSe@S-thick.

**Figure 2:**
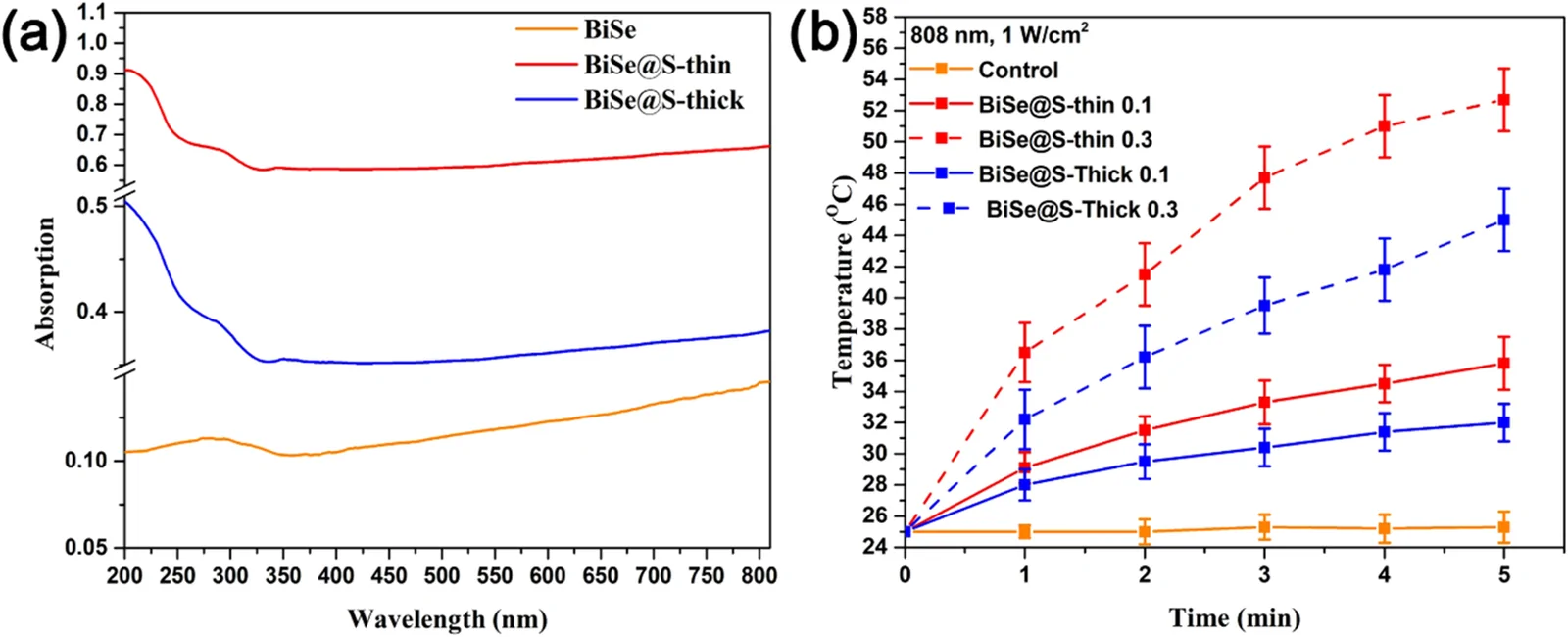
UV–Vis absorption spectra of silica-coated nanoplatelets with a thin (2–5 nm; BiSe@S-thin) and thick (50–80 nm; BiSe@S-thick) silica shell, compared to pristine Bi_2_Se_3_ nanoplatelets. Measurements were performed in aqueous suspension at a concentration of 0.1 g/L (a). Temperature increase of the silica coated nanoplatelets suspension in cell medium with concentrations 0.1 and 0.3 g/L compared to the control (cell medium) under 808 nm laser light irradiation with power or 1 W/cm^2^ (b). Temperature increase of nanoplatelets dispersed in HeLa cell culture medium under irradiation with an 808 nm laser (*P* = 1 W cm^–2^) over a period of 5 min (b).

In PTT, the objective is not only to generate heat but to control the temperature within a defined therapeutic window that maximizes tumor damage while minimizing injury to surrounding healthy tissue. PT-induced hyperthermia is typically categorized into mild (41–43 °C), subcoagulative (43–55 °C), and coagulative (>55 °C) regimes, with the subcoagulative range generally considered optimal for inducing apoptotic cell death while limiting inflammation and collateral damage.^4,77^

Figure 2b shows the PT performance of the BiSe@S-thin and BiSe@S-thick samples dispersed in the cell growth medium at a concentration of 0.1 and 0.3 g/L under laser illumination. The thermal response curves show a rapid and pronounced temperature increase in the silica-coated nanoparticle-containing cell medium compared to the control sample (cell medium), confirming efficient PT conversion under laser irradiation. Even at the lowest nanoplatelet concentration, the temperature rise was significantly higher than that of the control sample; however, the maximum temperature of the cell medium with nanoplatelets reached after 5 min remained below the threshold required for mild hyperthermia (≈41 °C). Although higher temperatures could in principle be achieved with higher laser power, power densities exceeding 1 W/cm^2^ are generally considered unsuitable for clinical PTT applications, as they exceed the maximum permissible exposure for the skin and increase the risk of permanent thermally induced tissue damage.^69–73^

The temperatures suitable for PT therapy (PTT)^4,77^ were achieved only for samples with the highest nanoparticle concentration, reaching 52 ± 3 °C for the BiSe@Si-thin sample and 45 ± 3 °C for the BiSe@Si-thick sample. These temperatures fall within the subcoagulative hyperthermia regime, where apoptotic cell death is typically induced, enabling effective tumor suppression while minimizing inflammation and damage to surrounding healthy tissue.^4,77^ Additionally, the obtained temperatures coincide with the biothermal threshold at which protein unfolding and DNA damage occur, typically reported in the range of 45–60 °C,^4,77^ further acknowledging the therapeutic potential of the silica-coated Bi_2_Se_3_.

To determine how the PT response translates into biological systems, subsequent in vitro assays were performed on a model cell line HeLa, including cell viability, apoptosis, and autophagy analyses after 5 min of laser irradiation in the presence of silica-coated Bi_2_Se_3_ nanoplatelets. Previous studies have confirmed that these silica-coated nanoplatelets exhibit negligible cytotoxicity for the HeLa cell system up to 0.5 g/L under identical conditions in the absence of irradiation.^38^ Therefore, any observed reduction in cell viability in this study can be attributed to the PT effect induced by laser irradiation.

As shown in Figure 3, while laser irradiation had no notable effect on cell viability (laser control), there was a clear reduction in the metabolic conversion of PrestoBlue dye after laser irradiation in samples with added silica-coated BiSe nanoparticles. We observed a statistically significant reduction in viability, dependent on both nanoparticle concentration and silica coating thickness, with higher concentrations and reduced thickness having a stronger effect. This was particularly notable with BiSe@Si-thin nanoplatelets, where a concentration of 0.3 g/L almost completely obliterated HeLa cell viability, while 0.1 g/L caused only a modest reduction (21% 4 h after irradiation and 11% 24 h after irradiation). It is worth mentioning that the assay measures viability indirectly through the metabolic activity of cells, and that lower viability 4 h after irradiation is most likely related to reduced metabolic activity of cells shortly after treatment and consequently thermal shock. These results are comparable to those reported for glycol-coated Bi_2_Se_3_ nanoparticles; however, the key advantage of our system lies in the use of a silica coating, which, as demonstrated, provides improved biocompatibility while maintaining effective PT performance. Overall, cell viability inversely correlated with the PT performance of the nanoplatelets (Figure 2b), with samples exhibiting stronger PT responses showing more pronounced reductions in HeLa cell viability. It is worth noting that although Bi_2_Se_3_ nanoplatelets are primarily recognized as photothermal agents, ROS generation under irradiation has been reported for bismuth-based nanomaterials^78^ and therefore their contribution to cell death cannot be entirely excluded.

**Figure 3:**
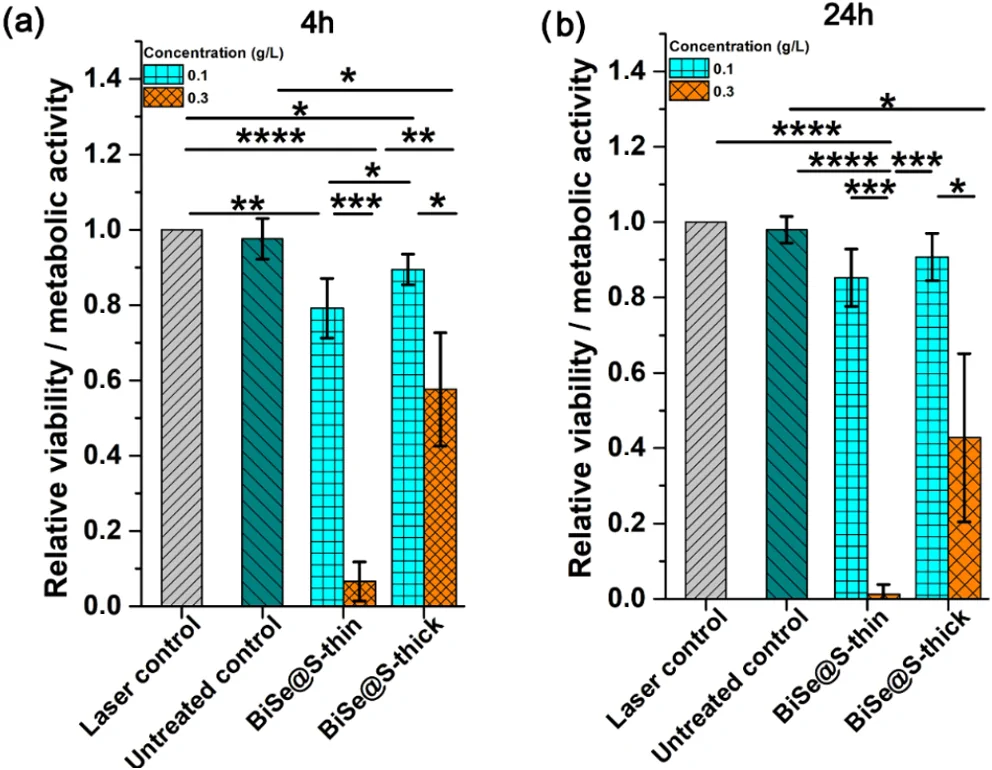
Cell viability of HeLa cell exposed to BiSe@Si-thin and BiSe@Si-thick nanoparticles. Cells were exposed to 0.1 or 0.3 mg/mL of nanoparticle suspension in the growth medium and irradiated with laser light at a power density of 1 W/cm^2^ for 5 min. At 4 h (A) or 24 h (B) postirradiation, cell viability was evaluated using the PrestoBlue Viability Assay. Obtained values were corrected for background fluorescence and normalized to laser control cells. Results are expressed as means ± SEM of four independent experiments. *p < 0.05, **p < 0.01, ***p < 0.001, and ****p < 0.0001.

The PT activity of silica-coated Bi_2_Se_3_ was further confirmed using an enzymatic apoptosis assay, which evaluated the activity of caspase-3/7 Apo-ONE Assay in HeLa cells irradiated with a laser. The results showed that activity of caspase-3/7 was significantly higher in the case when cells were exposed to silica-coated BiSe nanoplatelets and irradiated, compared to the control group (untreated control), while laser irradiation alone had no effect on apoptosis (Figure 4a). Interestingly, almost no apoptosis was detected in BiSe@Si-thin nanoparticles at a concentration of 0.3 g/L, where cell viability was lowest. As the Apo-ONE Assay relies on the activity of functional caspase-3 and -7, the very low substrate cleavage observed in this case may reflect extensive photothermal damage resulting in rapid loss of cellular integrity or the predominance of nonapoptotic cell death mechanisms. For example; severe thermal stress can shift cell death from caspase-dependent apoptosis toward necrotic cell death due to loss of cellular integrity and protein denaturation.^79,80^ However, additional studies would be required to confirm the basis of this observation.

**Figure 4:**
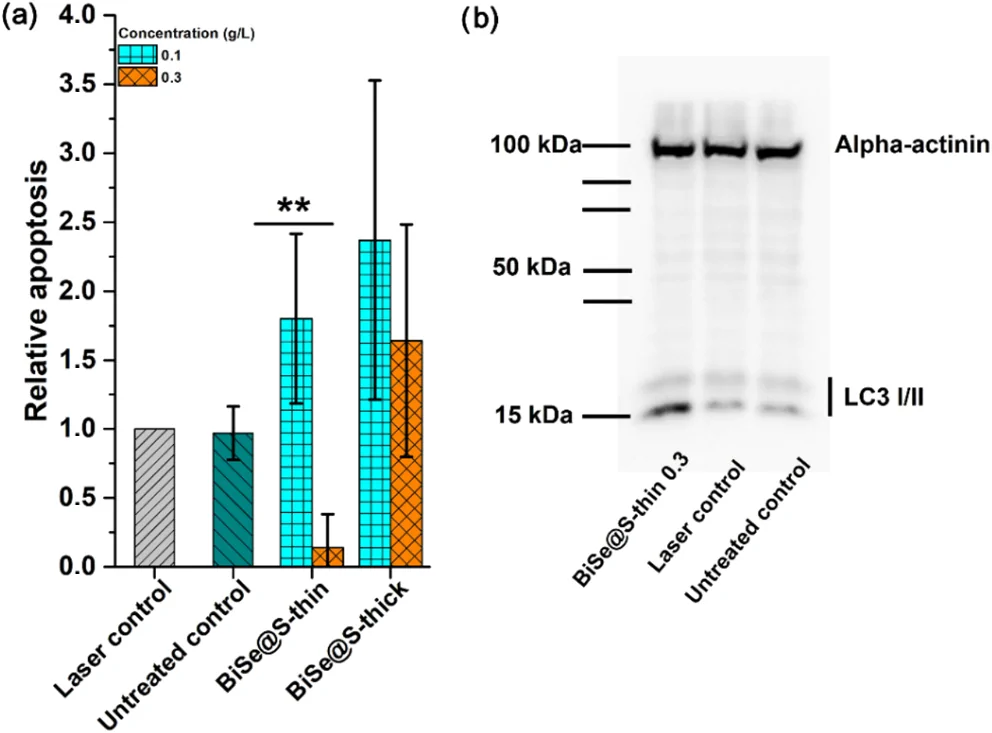
Apoptosis and autophagy-related responses in HeLa cell exposed to BiSe@Si-thin and BiSe@Si-thick nanoparticles. Cells were exposed to 0.1 or 0.3 mg/mL of nanoparticle suspension in the growth medium and irradiated with laser light at a power density of 1 W/cm^2^ for 5 min. At 24 h postirradiation, cells were evaluated for apoptosis using the Apo-ONE Homogeneous Caspase-3/7 Assay (a) or the cell lysates were subjected to Western blotting and probing for LC3 I/II protein (b). Obtained values in the apoptosis assay were corrected for background fluorescence and normalized to laser control cells. Results are expressed as means ± SEM of four independent experiments. **p < 0.01. (b) One representative experiment out of three is shown with α-actinin serving as a loading control.

Autophagy has been reported as one of the possible pathways responsible for the PT-dependent cell toxicity of Bi_2_Se_3_ and other nanoparticles.^36,81^ To do a preliminary investigation whether autophagy-associated responses were involved in the PT effect of silica-coated Bi_2_Se_3_ nanoplatelets, the presence of LC3-II protein was evaluated in HeLa cell lysates after thermal treatment, as the conversion of microtubule-associated proteins 1A/1B light chain 3B (LC3) from cytoplasmic LC3-I to autophagosomal LC3-II is an indicator of autophagy.^36,81^ Western blot analysis demonstrated that the amount of LC3-II was strongly increased in BiSe@Si-thin nanoparticle-exposed cells upon laser irradiation. At the same time, no such effect was observed in cells exposed only to laser irradiation, suggesting involvement of autophagy-associated pathways in HeLa cells upon laser irradiation of silica-coated Bi_2_Se_3_ nanoplatelets.

Lastly, we qualitatively analyzed the uptake of silica-coated Bi_2_Se_3_ nanoparticles in HeLa cells, as this is an important factor for efficient phototherapy. Several factors affect uptake, including the type, size, and surface charge of the nanoparticles used, as well as the characteristics of the target cells.^82–84^ We performed a qualitative immunofluorescence experiment by exposing HeLa cells to 0.2 mg/mL BiSe@Si-thin and BiSe@Si-thick nanoparticles, both modified with silane-functionalized fluorescein molecules. After 2 and 6 h of internalization, cells were fixed and counterstained with a nuclear DAPI stain. Bright-field images show clustering of silica-coated Bi_2_Se3 nanoplatelets within HeLa cells (Figure 5a,b, left panels). Clusters remained after intensive washing and cell manipulation during the immunofluorescence protocol, indicating that silica-coated Bi_2_Se_3_ nanoplatelets can attach to HeLa cells. Qualitative visual assessment shows that the number of attached nanoplatelets was considerably higher for BiSe@Si-thin at both 2 and 6 h after exposure compared to BiSe@Si-thick. When analyzing the cell nucleus focal planes by fluorescence microscopy (Figure 5, middle and right panels), small speckles of green nanoparticles were observed in HeLa cells, suggesting that a small proportion of Bi2Se3 nanoparticles can be internalized. Internalization was more efficient with BiSe@Si-thin nanoparticles, which were located predominantly in the cytoplasm and perinuclear region (Figure 5b, right panels). BiSe@Si-thick nanoplatelets were observed much less frequently, suggesting reduced internalization, most likely due to increased size. We observed that prolonged exposure (up to 6 h) did not increase total nanoparticle accumulation compared to a 2 h exposure (Figure 5), suggesting that internalization is occurring soon after exposure. However, we cannot exclude the possibility of nanoparticle aggregation on the cell surface, which may prevent long-term internalization and intracellular accumulation.

**Figure 5:**
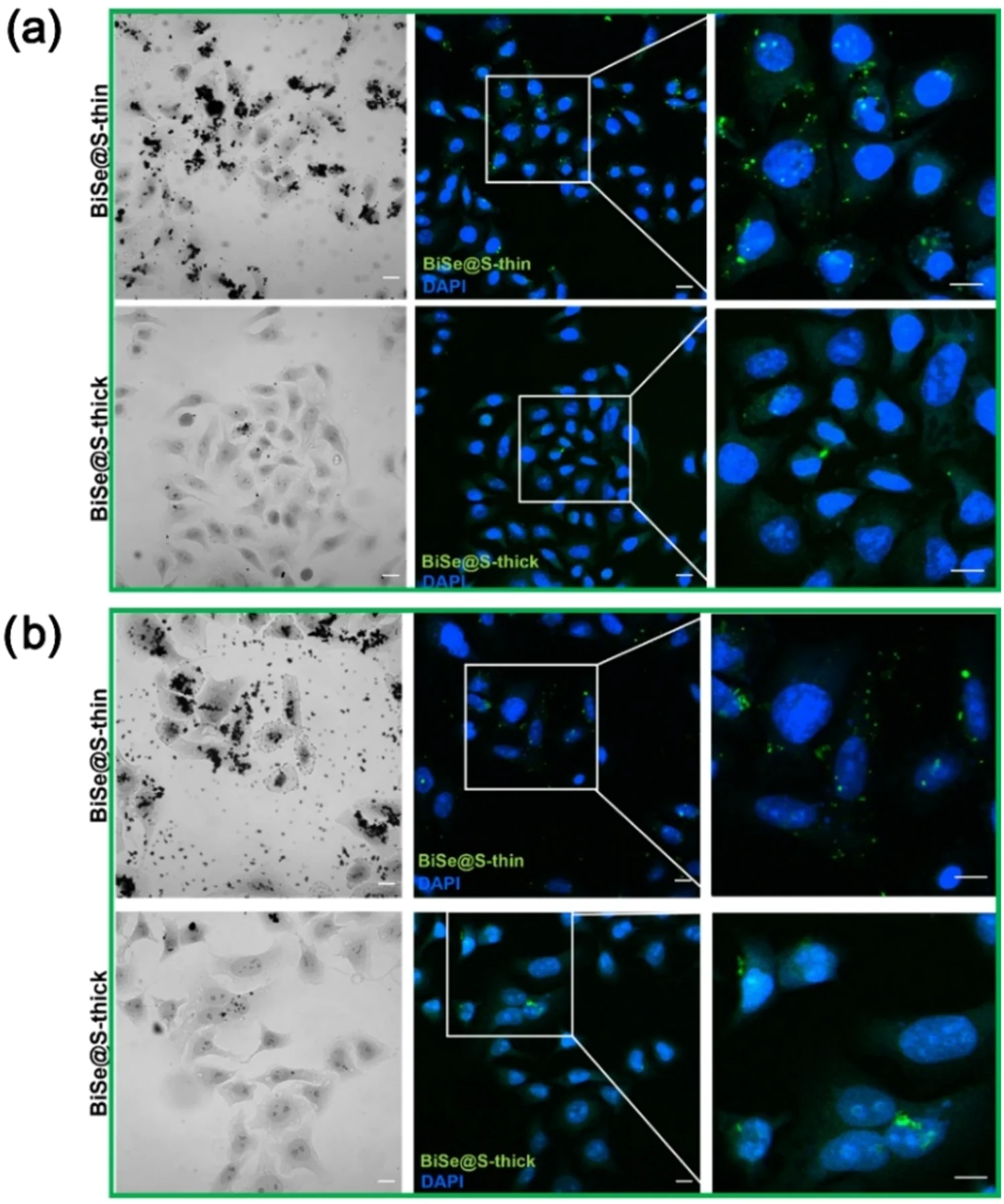
Internalisation of fluorescein-functionalized BiSe@Si-thin and BiSe@Si-thick nanopartilces in HeLa cells. Cells were exposed to 0.2 mg/mL of nanoparticle suspension in the growth medium for 2 h (a) and 6 h (b). Cells were the fixed and stained with DAPI. Representative images show cells in bright field (left panels) and fluorescein-functionalized BiSe@Si-thin (green) and DAPI-stained nuclei (blue) in the middle and right panels. The micrographs are representative and show the midcell body/nucleus focal planes. Scale bar = 10 μm.

The cellular interaction results with nanoplatelets observed in this internalization study deviate from commonly reported trends in the literature. As the cell membrane is typically negatively charged, positively charged nanoparticles are generally considered more favorable for cellular attachment and uptake.^82,84–86^ However, despite their negative surface charge under physiological conditions, the BiSe@S-thin nanoplatelets exhibited pronounced attachment to the cell membrane, along with partial internalization. Considering qualitative assessment of internalization, the results and the fact that the silica-coated samples exhibit comparable surface charge at physiological pH (Figure 1f), the observed differences in internalization can be attributed to particle size rather than surface charge, highlighting its important role in cellular interactions and uptake in this system. However, according to the literature, full nanoparticle internalization is not required to achieve effective cell death, as both surface-associated and internalized nanoparticles contribute to the overall photothermal (PT) response. Moreover, the combined presence of nanoparticles at the membrane and within the intracellular space enhances the overall therapeutic effect.^87^

## Conclusions

This work reports an in vitro assessment of silica-coated Bi_2_Se_3_ nanoplatelets for PTT-related antitumor applications. The investigated silica-coated systems were evaluated as an alternative to glycol-coated Bi_2_Se_3_ nanoplatelets, which have previously been reported to exhibit less favorable biocompatibility and altered optical absorption characteristics.^74^ The results demonstrate that silica coating improves the suitability of Bi_2_Se_3_ nanoplatelets to be relevant for the biomedical applications due to retaining and enhancing the characteristic absorption associated with the Bi_2_Se_3_ core in the spectral region >400 nm, compared to pristine Bi_2_Se_3_ nanoplatelets. This is especially beneficial for PTT, since lower nanoparticle concentrations can be applied to achieve the same therapeutic effect. However, the increase in the silica layer does not lead to a proportional increase in absorption. Compared to the BiSe@Si-thin sample, the BiSe@Si-thick display decreased absorption, but was still superior to the pristine nanoplatelets. Both BiSe@Si-thin and BiSe@Si-thick nanoparticles promote efficient PT-induced cytotoxicity. At a concentration of 0.3 g/L, laser irradiation (1 W/cm^2^) generates temperatures within the subcoagulative hyperthermia regime, promoting controlled apoptotic cell death. Among the two systems, BiSe@Si-thin nanoparticles demonstrate better therapeutic performance, driven by higher PT conversion, and based on qualitative observations, enhanced cellular association and internalization. No intrinsic cytotoxicity is observed in the absence of irradiation, suggesting that cell death arises from the synergistic action of nanoparticles and laser exposure, i.e., it is PT-driven. Furthermore, cell qualitative internalization studies indicate that particle size can be an influencing factor compared to surface charge, governing cellular uptake, with smaller, thin-silica-coated nanoplatelets exhibiting higher internalization efficiency than their thick-silica-coated counterparts. While the present study demonstrates that silica-coated Bi_2_Se_3_ nanoplatelets are a promising agent for photothermal applications, their biological fate remains to be fully elucidated. Future studies should investigate nanoparticle stability in physiological media, uptake by immune cells, degradation kinetics, biodistribution, and clearance mechanisms, as well as the long-term effects of bismuth- and selenium-containing degradation products. Addressing these aspects will be critical for advancing these materials toward biomedical applications.

## Supplementary Material



## Notes

Additional experimental details and discussion regarding the colloidal stability and physicochemical properties of the nanoplatelets, including dynamic light scattering (DLS) measurements and time-dependent ζ-potential measurements (PDF)
