# Supplementary material for: In Vitro Evaluation of Silica-Coated Bi2Se3 Nanoplatelets as Photothermal Nanomaterials
Source: ACS Omega. 2026 Aug 5;11(32):48401–11. doi: 10.1021/acsomega.6c05034 (PMC13491952; doi:10.1021/acsomega.6c05034)
Supplement: Supplementary file 1 [file ao6c05034_si_002.pdf]

In Vitro Evaluation of Silica-Coated Bi_2Se_3 Nanoplatelets as Photothermal Nanomaterials

Blaž Belec[1]*, Martina Bergant Marušič[2], Sebastjan Nemec[1], Nina Kostevšek[3,4], Matjaž Valant[1]

[1] Materials Research Laboratory, University of Nova Gorica, Slovenia
[2] Laboratory for Environmental and Life Sciences, University of Nova Gorica, Slovenia
[3] Department for Nanostructured Materials, "Jožef Stefan" Institute, Ljubljana
[4] Jožef Stefan International Postgraduate School, Ljubljana, Slovenia

Supplemental Information

S1: DLS hydrodynamic particle size measurements

The time-dependent colloidal stability of the silica coated BiSe platelets was investigated in pure water and biological media using DLS hydrodynamic size measurements. The BiSe@S-thin and BiSe@S-thick platelets in dry powder form were dispersed in pure water up to a concentration of 0.3 mg/mL (the same concentration that was used in cell tests). The dispersion of the platelets in water was achieved using ultrasonication (3 x 10 s, intensity 40 %, VCX-500—110 Vibracell, Sonics, Newtown, CT, USA). The samples in biological media were prepared in a similar procedure as the aqueous sample. The silica coated platelets in dry powder form were dispersed in water to a concentration of 6 mg/mL using ultrasonication and then the obtained aqueous platelet suspension was diluted with either DMEM or DMEM supplemented with 10 % FBS to achieve the 0.3 mg/mL. The volume fraction of the aqueous platelet suspension diluted with biological media represented 5 % of the volume and in this way the composition of the biological media was not altered significantly. The DLS measurements were done on a Litesizer 500 particle analyzer (Anton Paar, Graz, Austria) in a proprietary polycarbonate Omega cuvette (Anton Paar, Graz, Austria). Measurements were done at 23 °C in triplicates.

The hydrodynamic size distribution of the BiSe@S-thin and BiSe@S-thick in water was in the range of sub-micron to several µm, indicating partial agglomeration (Figure S1). A similar hydrodynamic size is retained in biological media, however with a narrower size distribution. In the time frame of the measurements, the hydrodynamic size distribution did not change significantly, except for the BiSe@S-thick sample in DMEM + 10 % FBS, where a larger particle population become present. Taken together, the DLS hydrodynamic particle size measurements of the platelets in biologic media show that the complex composition of the biological media consistent of high concentrations of inorganic salts,

organic molecules, and proteins (in FBS) do not lead to significant increase of agglomeration of the platelets in the experiment timeframe.

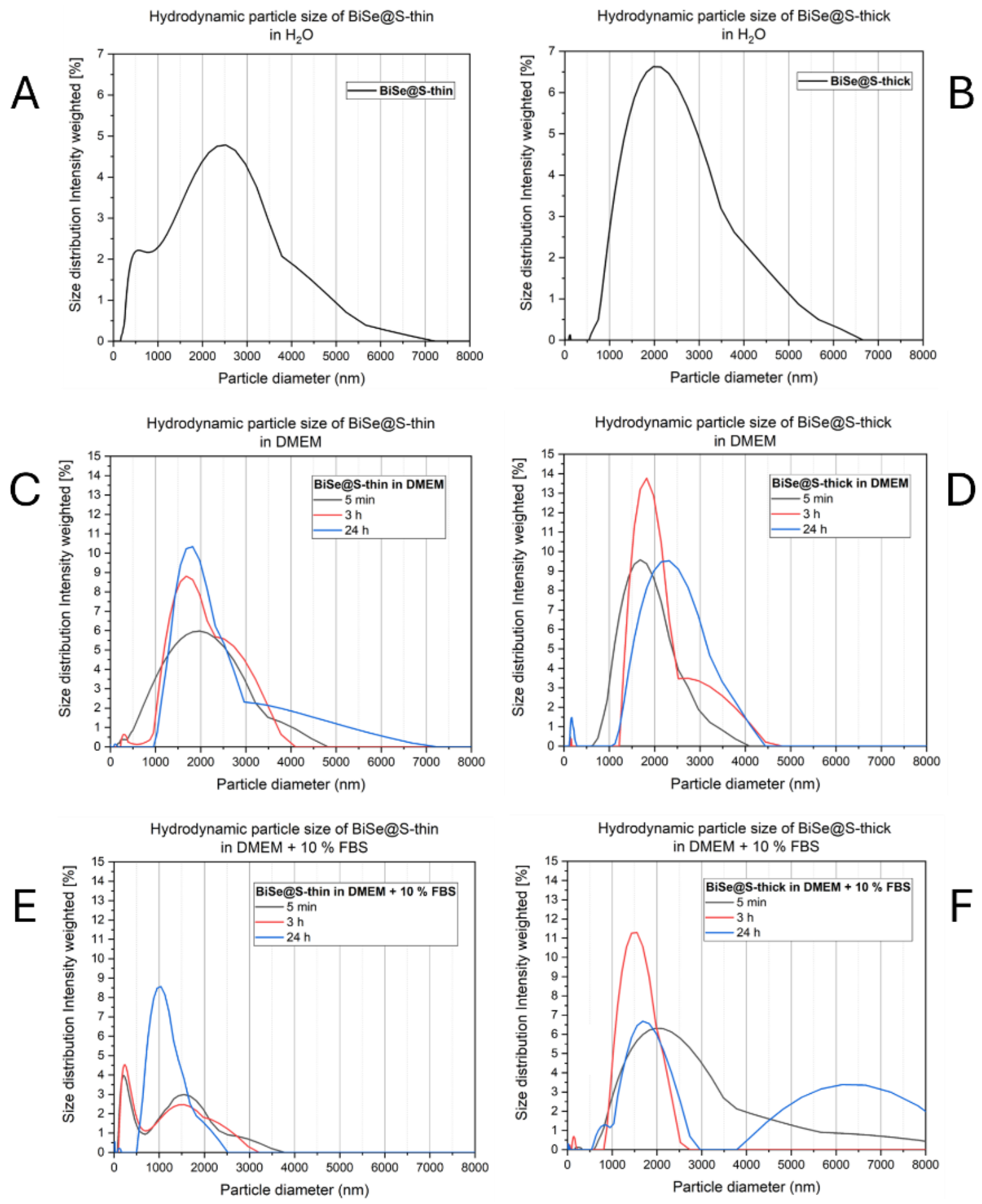

F***igure S1***: DLS hydrodynamic particle size measurements of BiSe@S-thin and BiSe@S-thick in H_2O (**A**, **B**) and in biological media DMEM (**C**, **D**) and DMEM supplemented with 10 % FBS (**E**, **F**) at different time points.

S2: Time dependant ζ-potential measurements

The time-dependent colloidal stability of the silica-coated platelets was also additionally investigated using ζ-potential measurements by measuring the electrophoretic mobility of the silica-coated platelets using the same apparatus as for DLS measurements (described in S1). The samples for ζ-potential measurements were prepared using the same procedure as for DLS measurements (described in S1), and all ζ-potential measurements were done at particle concentration 0.3 mg/mL.

ζ-potential measurements (**Figure S2**) show that both BiSe@S-thin and BiSe@S-thick have a high negative ζ-potential in pure water of -26.4 ± 0.6 mV and -27.4 ± 0.7 mV, respectively. After dilution in biological media, the ζ-potential is lowered. For the BiSe@S-thin the ζ-potential after 24 h in suspension with DMEM is retained at -14.2 ± 0.2 mV and with DMEM supplemented with 10 % FBS is

-11.0 ± 0.6 mV. For the BiSe@S-thick sample the ζ-potential after 24 h in DMEM is -11.3 ±0.1 mV and in DMEM supplemented with 10 % FBS is -11.7 ±0.1 mV. No explanation was found why the ζ-potential potential at 5 min and 3 h after mixing the BiSe@S-thick with DMEM supplemented with 10 % FBS was around zero, when in all other cases was not equal to zero. Possible instrumentation error could not be excluded. However, the measurement after 24 h shows retained ζ-potential in a similar degree as with the other samples.

A general lower zeta potential of the silica-coated BiSe platelets compared to aqueous suspension is expected. The surface charge is partially masked by the adsorption of biological media components, such as inorganic salts, organic compounds, and proteins. However, the retained ζ-potential zeta potential of the platelets in the biological media is significant and contributes to the colloidal stability of the platelets in biological media and during experiments involving cells.

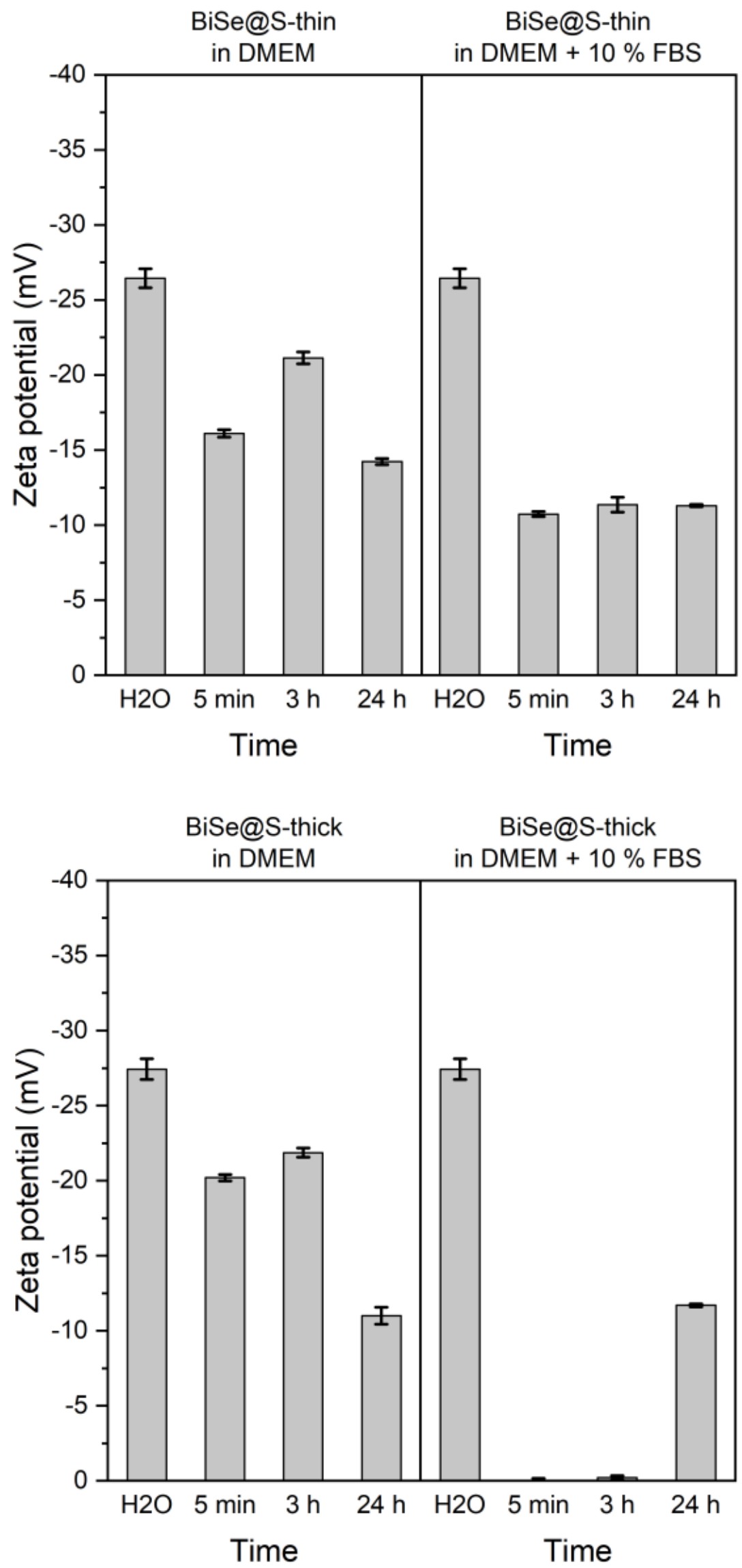

Figure S2: ζ-potential measurements of silica-coated BiSe platelets in H_2O and biological media at different time points.
